# The conflict between need and fear: how privacy concerns moderate the influence of depression on university students’ acceptance of AI music therapy

**DOI:** 10.3389/fpsyg.2026.1768759

**Published:** 2026-02-24

**Authors:** Yang Zhu, Riming Liu

**Affiliations:** 1 College of Arts and Media, Tongji University, Shanghai, China; 2 College of Humanities, Tongji University, Shanghai, China

**Keywords:** AI music therapy, depression, digital mental health, moderation effect, privacy calculus, technology acceptance (TAM)

## Abstract

**Background:**

AI-driven music therapy offers a promising, accessible digital intervention for the growing mental health crisis in universities. The “Deficiency Compensation Hypothesis” suggests that depression may drive students toward such digital help-seeking. However, the inherent data sensitivity of AI tools triggers the “Privacy Calculus,” potentially inhibiting adoption. This study investigates the interplay between depression severity, privacy concerns, and the intention to use AI music therapy among university students.

**Methods:**

A cross-sectional survey was conducted with 612 university students in China. The study measured depression levels (PHQ-8), AI-specific privacy concerns, perceived usefulness, and intention to use. A hierarchical regression model with moderation analysis was employed to examine whether privacy concerns weaken the association between distress and help-seeking motivation.

**Results:**

Participants exhibited mild depression on average (PHQ-8 Mean = 6.07). Regression analysis revealed that depression positively predicted the intention to use AI music therapy (*β = 0.128, p < 0.001*), supporting the distress-driven help-seeking hypothesis. Crucially, privacy concerns acted as a significant negative moderator (*β = −0.086, p = 0.015*). Simple slope analysis indicated that the motivating effect of depression on usage intention was significant only for students with low privacy concerns but was nullified in those with high privacy concerns.

**Conclusion:**

The findings highlight a critical paradox in digital mental health: while depressive symptoms are positively associated with students’ intention to seek AI-based help, privacy fears can significantly attenuate this association. For highly privacy-sensitive individuals, the need for therapeutic relief is overridden by the fear of surveillance. Consequently, developers and universities must prioritize “privacy by design” and transparent trust mechanisms, rather than relying solely on algorithmic precision, to ensure these tools can serve as effective emotional support for vulnerable students.

## 1 Introduction

### 1.1 Research background

#### 1.1.1 The mental health crisis in the digital age

In recent years, the mental health of university students has become a growing concern worldwide (Ferrari et al., 2022). A report from the World Health Organization (WHO) indicates that depression has become one of the leading causes of disability and death among adolescents. In China, the Ministry of Education and several epidemiological surveys show a year-on-year increase in the detection rate of depression among university students, with some regions exceeding 20% (Lin and Li, 2025). However, faced with this massive demand, the traditional mental health service system is confronted with severe challenges. On one hand, there is a serious shortage of psychological counselors in universities, making it difficult to cover all students in need (Juniar et al., 2025). On the other hand, constrained by traditional cultural norms and stigma, many students suffering from depressive symptoms would rather endure their pain alone than step into an offline counseling office (Li et al., 2024). This situation, characterized by both a supply–demand imbalance and barriers to seeking help, urgently calls for the emergence of new intervention models.

#### 1.1.2 The rise and advantages of AI music therapy

With breakthroughs in Generative AI technology, AI-empowered psychological therapy tools have emerged (Fitzpatrick et al., 2017; Ni and Jia, 2025). Among them, AI music therapy has attracted considerable attention due to its unique advantages. A large body of research has already confirmed the effectiveness of traditional music therapy in alleviating stress and depressive symptoms (De Witte et al., 2022; Liu et al., 2025). Unlike the passive listening of traditional music, modern AI systems (such as Suno, Mubert, etc.) can generate personalized therapeutic music in real-time based on the user’s emotional state, heart rate variability (HRV), or even brainwave data (Liu et al., 2024). For university students with depression, AI music therapy holds a natural appeal: first, its low barrier to entry and high accessibility allow students to access services anytime and anywhere with just a smartphone, breaking through the time and space constraints (Gbollie et al., 2023); second, the sense of security from depersonalized interaction helps avoid the evaluation anxiety present in interpersonal communication, providing a safe “psychological buffer zone” for depressed individuals with social withdrawal tendencies (Crawford et al., 2024). Therefore, AI music therapy is considered a potentially effective path to mitigating the campus mental health crisis (He et al., 2024). Specifically for depressed students, who often experience anhedonia (the inability to feel pleasure) and motivational deficits, the sensory and non-verbal nature of music offers a unique therapeutic modality that is less cognitively demanding than traditional talk-based therapies. It provides an alternative channel for emotional expression and regulation, making it a theoretically relevant intervention for this population.

#### 1.1.3 Privacy risks and acceptance challenges

However, technological development also brings corresponding challenges and risks. The high degree of personalization in AI music therapy relies on the deep mining of users’ sensitive data. To generate precise musical prescriptions, algorithms often need to collect highly private information such as users’ emotional records, physiological indicators, and even voice intonation. This collection of affective and physiological data is qualitatively different and potentially more intrusive than the text-based disclosures common in AI chatbots, raising unique privacy concerns. In the context of price discrimination based on big data analysis and frequent data breaches, this “panoptic surveillance” of one’s inner mental world has triggered widespread privacy anxiety (Barth and de Jong, 2017). For university students with depressive tendencies, who are already sensitive and suspicious, will this privacy concern become a key hindering factor? Specifically, because the data captured by AI music therapy (e.g., emotional, vocal, physiological) can be perceived as more revealing of one’s inner state than text-based data, understanding the role of privacy concerns becomes particularly critical. This is a pressing real-world question that needs to be answered (Li et al., 2024).

### 1.2 Research questions and objectives

Although AI music therapy offers a new path to address the escalating campus mental health crisis, its promotional success hinges on a core psychological trade-off: to what extent are potential users (especially high-risk depressed groups) willing to risk privacy breaches in exchange for emotional comfort? This trade-off is the central logic of Privacy Calculus Theory (PCT), which posits that individuals weigh perceived benefits against perceived costs (privacy risks) when deciding whether to disclose information. However, this framework is often underutilized in technology acceptance studies concerning mental health. Existing research on the Technology Acceptance Model (TAM) often focuses on functional attributes of a system (e.g., usefulness, ease of use), while paying less attention to how users’ own psychological traits (such as depressive state) act as a proxy for the ‘perceived benefit’ in the privacy calculus (Kleine et al., 2025). On the other hand, while research on privacy concerns is mature, it has mostly concentrated on the e-commerce domain (Xu et al., 2009), and its mechanism of action in a highly sensitive context like mental health remains unclear.

Based on this, this study proposes the following core research questions:

Driving Mechanism: Does the level of depression among university students positively predict their intention to use AI music therapy? (i.e., verifying the logic of “distress-driven help-seeking”).

Boundary Condition: Do privacy concerns moderate the relationship between depression and intention to use? (i.e., verifying the boundary of “privacy-hindered help-seeking”).

### 1.3 Significance of the study

#### 1.3.1 Theoretical significance

This study constructs an integrated analytical framework by introducing a psychopathological variable (depression) into the classic Technology Acceptance Model (TAM) and combining it with Privacy Calculus Theory. This not only extends the applicability of TAM to special populations (psychologically sub-healthy individuals) and specific contexts (digital healthcare) (Zhang et al., 2025) but also enriches our understanding of the “privacy paradox” in the field of health behavior (Barth and de Jong, 2017). More importantly, by identifying a key psychological boundary condition—privacy concern—this study specifies when psychological distress is more or less likely to translate into the intention to seek digital help. This moves beyond simply extending existing models and offers a more nuanced explanation for the complex decision-making process of vulnerable users, highlighting a critical point of friction in the adoption of digital mental health tools.

#### 1.3.2 Practical significance

The findings of this study will directly serve the digital transformation of mental health services in universities. If privacy concerns are confirmed to be a key inhibiting factor, then universities and developers, when promoting AI tools, cannot simply focus on publicizing “powerful features” but must front-load “data security commitments.” The conclusions will provide strategic guidance not only for designing AI products that are more humanistic, but also for university health administrators to formulate transparent data governance policies, ensuring that digital interventions are both accessible and trustworthy.

## 2 Literature review

### 2.1 Theoretical foundations

#### 2.1.1 Technology acceptance model (TAM) and its evolution

The Technology Acceptance Model, first proposed by Davis (1989), is one of the most influential theoretical models for explaining users’ behavioral intentions to adopt information systems. Rooted in the Theory of Reasoned Action (TRA), TAM’s core proposition is that an individual’s Behavioral Intention to use a technology is primarily determined by two core beliefs:

Perceived Usefulness (PU): The degree to which a user believes that using a particular system will enhance their job performance or quality of life.

Perceived Ease of Use (PEOU): The degree to which a user believes that using the system will be free of effort.

In the mental health domain, TAM has been widely applied to explain the adoption of online counseling, mental health apps, and wearable devices (Gbollie et al., 2023; Kleine et al., 2025). However, the traditional TAM model assumes users are perfectly rational decision-makers, often overlooking the distorting effect of emotional states (such as anxiety, depression) on cognitive evaluations, and it does not fully consider external social environmental factors.

To address the shortcomings of the TAM model, Venkatesh et al. (2003) proposed the Unified Theory of Acceptance and Use of Technology (UTAUT). UTAUT integrates eight major models, including TAM, and proposes four core determinants: Performance expectancy, effort expectancy, Social Influence, and Facilitating Conditions. “Social Influence” refers to the degree to which an individual perceives that important others (such as friends, classmates, teachers) believe they should use the new technology. “Facilitating Conditions” refers to the individual’s perception of the organizational and technical resources available to support the use of the system. In recent years, numerous studies have applied UTAUT to explain the acceptance of AI chatbots or tools (Li et al., 2024; Qi et al., 2025).

For university students, these two new variables in the UTAUT model undoubtedly have strong explanatory power. For instance, recommendations from peers (Social Influence) or whether the university’s counseling center officially introduces and provides technical support (Facilitating Conditions) could significantly impact the adoption of AI music therapy.

Although the UTAUT model offers a more comprehensive explanatory framework, this study opts for a more parsimonious model. Considering that the core objective of this study is to deeply analyze the internal conflict between an individual’s psychopathological state (depression) and their perception of privacy risks, rather than external environmental factors, we have chosen a more streamlined model focused on core psychological variables. This study treats the core belief of the classic TAM (Perceived Usefulness) as an important control variable, thereby focusing the research on the non-rational emotional factor of depression. This allows for a clearer examination of the interplay between “deficiency compensation” and “privacy calculus.” We acknowledge this is a simplified perspective and suggest in the limitations section that future research should integrate the internal psychological mechanisms of this model with the external environmental factors of UTAUT to construct a more complete model of digital mental health intervention acceptance for university students (Yang et al., 2024).

#### 2.1.2 Privacy Calculus Theory

With the development of big data technology, users face increasingly severe privacy risks when using digital services. The Privacy Calculus Theory, proposed by Culnan and Armstrong (1999), posits that individuals conduct a rational “cost–benefit analysis” when disclosing personal information (Xu et al., 2009).

Benefits: Include personalized services, convenience, economic rewards, or (in the context of this study) psychological comfort (Chaudhry and Debi, 2024).

Costs (Risks): Primarily the potential risks arising from privacy breaches, such as social discrimination (stigma), identity theft, or a sense of being monitored (Li et al., 2024).

The theory suggests that users will choose to relinquish their privacy only when the perceived benefits outweigh the perceived risks. In the context of AI music therapy, students face a typical privacy calculus trade-off: on one hand, they desire the emotional relief brought by AI (benefits), and on the other, they fear that the leakage of their depression data could lead to academic setbacks or social exclusion (costs). This trade-off may be even more intense in depressed populations, as they are more sensitive to negative evaluations.

### 2.2 Key variables and hypotheses

#### 2.2.1 Depression and intention to use AI music therapy: the Deficiency Compensation Hypothesis

The Deficiency Compensation Hypothesis provides the primary theoretical lens for H1. This hypothesis suggests that when individuals’ needs in real life (such as social interaction, emotional support) are not met, they tend to turn to online environments to seek alternative forms of satisfaction (McKenna and Bargh, 2014).

This hypothesis has been validated in multiple fields. For example, research shows that individuals with high levels of social anxiety are more inclined to self-disclose on the internet because the anonymous online space reduces their fear of negative evaluation (Sheldon, 2009). In the field of health communication, this effect is particularly pronounced. Patients with socially stigmatized diseases (such as HIV, schizophrenia) are more likely to seek information and emotional support in anonymous online health communities due to fear of real-world discrimination (Wright and Bell, 2003).

We extend this logic to the context of our study. For depressed university students, traditional face-to-face help-seeking is fraught with resistance, whereas AI music therapy, through its unique interactive features, provides precise “compensation” for these barriers (Potts et al., 2025). Specifically:

In the face of the fear of negative evaluation that traditional counseling might trigger, AI offers a completely non-judgmental interactive environment.

Addressing the concern of identity exposure due to stigma, the anonymous nature of AI provides a safe psychological space.

For individuals who find it difficult to initiate offline help-seeking due to social anxiety and energy depletion, the on-demand accessibility and depersonalized nature of AI tools significantly lower the barrier to seeking help.

In summary, these features of AI music therapy precisely meet the contradictory needs of depressed individuals who both crave help and fear interpersonal contact. Several empirical studies indirectly support this view, indicating that adolescents with higher levels of depression use the internet more frequently as an emotion regulation tool (Sun et al., 2025). Therefore, we have ample reason to infer that when real-world help-seeking channels are obstructed, the severity of depressive distress will act as a powerful driving force, prompting individuals to turn to this low-social-pressure, high-security digital form of help-seeking.

It is important, however, to acknowledge an alternative perspective. From a classic Technology Acceptance Model (TAM) viewpoint, depressive symptoms—often associated with cognitive fatigue, motivation, and reduced self-efficacy—could plausibly be interpreted as a factor that reduces perceived ease of use or increases perceived effort, thereby acting as a barrier to technology adoption. While acknowledging this possibility, our study focuses on the motivational aspect, conceptualizing depression as a ‘need’ that drives help-seeking in a low-stigma, non-interpersonal context. We hypothesize that for digital tools that bypass social barriers, the compensatory ‘pull’ of need will outweigh the ‘push’ of cognitive barriers, a premise we test with H1.

Based on this, we propose hypothesis H1:

*H1*: After controlling for variables such as perceived usefulness, university students’ level of depression will significantly and positively predict their intention to use AI music therapy.

#### 2.2.2 The moderating role of privacy concerns: boundaries of the privacy paradox

Based on the logic of Privacy Calculus Theory (PCT), although depression may drive the intention to seek help, this process is not unconditional. Privacy Concern refers to an individual’s worries about the improper collection, use, or disclosure of personal information. In the digital health domain, health data (especially mental health data) is considered the most sensitive category of privacy (Kasen et al., 2024). For depressed students, the consequences of a data breach are not just nuisance calls but could lead to severe social exclusion and reputational damage.

Privacy Calculus Theory suggests that privacy concerns can inhibit the process of translating “need” into “action.” Existing research has shown that privacy concern is a significant factor influencing users’ adoption of AI chatbots for mental health (Li et al., 2024). Specifically:

Low Privacy Concern Context: When students perceive the AI system as secure or do not mind their data being collected, the distress from depression will directly translate into a strong motivation to seek help (consistent with the logic of H1). In this case, the more severe the depression, the higher the intention to use.

High Privacy Concern Context: When students are extremely worried about data leakage, this fear activates a “defense system.” Even if they are very depressed and in great need of help, the potential catastrophic consequences of a privacy breach (e.g., being advised to withdraw from school, being gossiped about by peers) will override the desire to seek help. At this point, the facilitating effect of depression on the intention to use will be offset by this “brake.”

That is, privacy concern is not just a direct negative factor but also a moderator that alters the strength of the relationship between depression and intention to use.

Based on this, we propose hypothesis H2:

*H2*: Privacy concern negatively moderates the relationship between depression level and intention to use. That is, as privacy concern increases, the positive predictive effect of depression on intention to use will gradually weaken.

### 2.3 Research model construction

**Figure 1:**
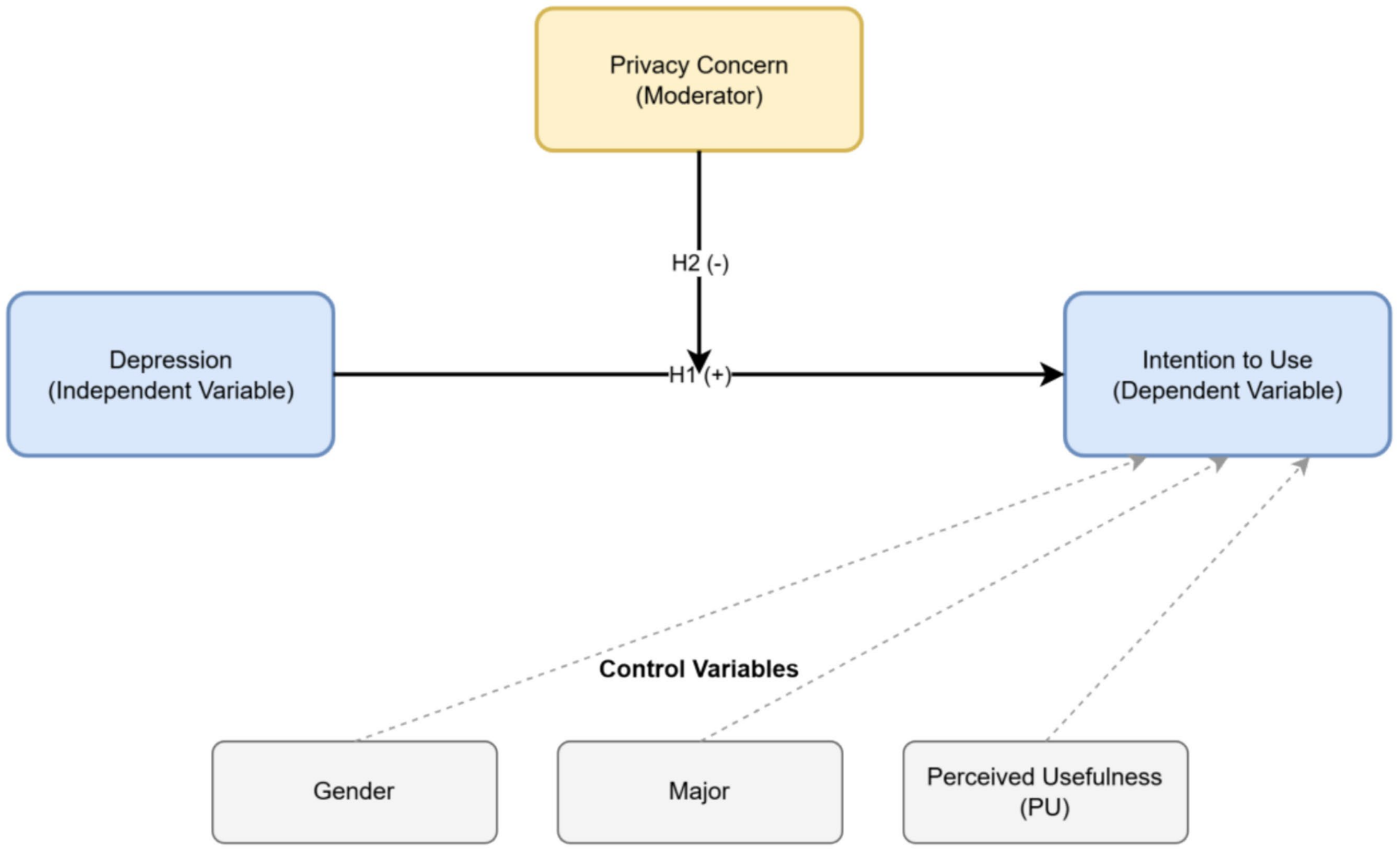
The proposed conceptual model.

Based on the theoretical deductions above, this study constructs a moderated effect model to deeply deconstruct the interplay between “distress (depression)” and “fear (privacy)” in the process of university students’ acceptance of AI music therapy. The conceptual framework of this model is shown in Figure 1.

Specifically, the model tests the main effect of depression severity (independent variable) on university students’ intention to use AI music therapy (dependent variable) (Hypothesis H1). Building on this, the model further explores whether privacy concern (moderator) plays a negative moderating role in the aforementioned main effect relationship (Hypothesis H2).

Furthermore, to rule out potential confounding influences, this study includes gender, major, age, and a core variable from the Technology Acceptance Model, Perceived Usefulness (PU), as control variables.

## 3 Methodology

### 3.1 Research design and procedure

This study employed a cross-sectional survey design to collect quantitative data through a structured questionnaire, aiming to validate the relationships among depression level, privacy concerns, and the intention to use AI music therapy.

#### 3.1.1 Questionnaire design and ethical considerations

The questionnaire consisted of three parts:

Informed consent and ethical statement: The first page of the questionnaire clearly informed participants of the research purpose, data anonymity, the voluntary nature of participation, and their freedom to withdraw at any time. It was specifically emphasized that all data would be used for academic statistical analysis only and would not involve personal identification, to minimize participants’ concerns.

Core Psychological Scales: Included measures for depression, privacy concerns, and technology acceptance.

Demographic Information: Included gender, age, grade level, major, and level of education.

#### 3.1.2 Sampling and data collection

The research subjects were university students (including undergraduates and postgraduates) in mainland China. Data was collected from March to April 2025. A combination of convenience sampling and snowball sampling was used to distribute the questionnaire link via online platforms like Wenjuanxing. To ensure sample diversity, the researchers distributed the questionnaire in student communities of comprehensive universities, science and engineering universities, and arts universities. To control data quality, the survey system had the following screening mechanisms, and manual cleaning was performed after data export:

IP Restriction: Each IP address was limited to one response to prevent duplicate submissions.

Patterned Response Detection: Invalid questionnaires with identical answers to all questions or showing obvious patterns were manually removed.

Response Time Control: Questionnaires completed in less than 90 s were excluded.

### 3.2 Participants

A total of 680 raw questionnaires were collected. After strict cleaning based on the above criteria, 612 valid samples were obtained, yielding an effective response rate of 90.0%.

The demographic characteristics of the sample are shown in Table 1.

**Table 1:** Demographic characteristics of the participants (*N* = 612).

Category	Sub-category	Frequency (*N*)	Percentage (%)
Gender	Male	264	43.1%
	Female	348	56.9%
Major	STEM	171	27.9%
	Humanities & Social Sci.	154	25.2%
	Arts & design	145	23.7%
	Medicine	142	23.2%

Gender: Female participants were in the majority, with 348 individuals (56.9%); there were 264 male participants (43.1%).

Major Background: The sample covered a diverse range of academic backgrounds, with STEM being the largest group (27.9%), followed by Humanities & Social Sciences (25.2%), Arts & Design (23.7%), and Medicine (23.2%).

Age: Participants were primarily young university students, with an average age of approximately 20.

### 3.3 Measures

The scales used in this study are all mature scales widely used domestically and internationally, adapted as necessary to the specific context of AI music therapy. Except for demographic variables, all items used a Likert 5-point scale (1 = Strongly disagree, 5 = Strongly agree), with the exception of the PHQ-8. The complete survey questionnaire used in this study can be found in Supplementary material A.

#### 3.3.1 Operational definition of the core construct: the AI music therapy scenario

Considering that “AI music therapy” is a cutting-edge and diverse concept, to ensure all participants had a consistent understanding of the core research object, we provided a clear scenario description before the relevant sections of the questionnaire. This definition aimed to highlight its core features of “automation” and “personalization” while excluding the confounding factor of human intervention. The specific description was as follows:

[Scenario Description] Please read carefully:

Imagine that your university counseling center or an app store has launched an “AI Emotion Regulation Assistant.”

It does not involve any human counselors.

It analyzes your state (e.g., voice intonation, heart rate, or self-report) through an AI algorithm and automatically generates personalized soothing music for you.

The purpose is to help you relax when you are stressed or in a bad mood.

Through this standardized description, we operationally defined the research object as a personalized music generation service that requires no human interaction, is driven by algorithms, and aims at emotion regulation, thereby ensuring the validity of the construct measurement.

#### 3.3.2 Scale content and adaptation process

Depression Severity: Measured using the mature PHQ-8 (Patient Health Questionnaire-8) scale (Kroenke et al., 2009). This scale includes 8 items (e.g., “Feeling down, depressed, or hopeless”), scored from 0 to 3, and has good reliability and validity in non-clinical populations. It was used directly in this study without adaptation.

Privacy Concern: Adapted from the classic mobile internet privacy concern scale by Xu et al. (2009). The adaptation process was mainly contextualization: we specified the general term “personal information” in the original scale to “emotional data” and “mental health status data” in our research context. For example, the original item “I am concerned that my personal information might be used improperly” was adapted to “I am concerned that my mental health status data could be disclosed to the university or third parties.” This adaptation aimed to enhance the relevance of the items to the research context.

Intention to Use & Perceived Usefulness: Adapted from the corresponding scales in the UTAUT model by Venkatesh et al. (2003). The adaptation was also contextual, replacing “the system” in the original items with “this type of AI tool” or “this AI music tool.” For example, “I intend to use the system” was adapted to “If I have the opportunity, I am willing to try this AI music tool.” Similar adaptations are commonly used in other AI acceptance studies (Li et al., 2024; Qi et al., 2025).

#### 3.3.3 Reliability and validity tests of the measurement model

To ensure the quality of the measurement tools, this study conducted reliability and validity tests.

Reliability Test: This study used Cronbach’s *α* coefficient to assess the internal consistency reliability of each scale. The specific results will be reported in Chapter 4.

Construct Validity Test: Since the classic Confirmatory Factor Analysis (CFA) has extremely strict model requirements, and considering the core purpose of this study was to validate the dimensional structure of the adapted scales, we employed Exploratory factor analysis (EFA) as a rigorous and widely accepted alternative to test the construct validity of the scales.

First, the data suitability test results showed a KMO value of 0.765 (greater than 0.7), and the Bartlett’s test of sphericity was significant (*χ*^2^(36) = 1991.84, *p < 0.001*), indicating that the data was very suitable for factor analysis.

Subsequently, we conducted an EFA on the 9 items of Perceived Usefulness, Privacy Concern, and Intention to Use, using Maximum Likelihood extraction and Varimax Rotation. The results clearly identified three factors, with the factor loading matrix shown in Table 2.

**Table 2:** Exploratory factor analysis (EFA) loadings for measurement items.

Item	Construct	Factor 1 (intention to use)	Factor 2 (privacy concern)	Factor 3 (perceived usefulness)
BI_1	Intention to use	0.815	−0.109	0.195
BI_2	Intention to use	0.803	−0.090	0.187
BI_3	Intention to use	0.798	−0.107	0.228
Priv_1	Privacy concern	−0.106	0.779	0.029
Priv_2	Privacy concern	−0.085	0.722	−0.022
Priv_3	Privacy concern	−0.062	0.730	0.008
PU_1	Perceived usefulness	0.145	0.021	0.636
PU_2	Perceived usefulness	0.174	−0.024	0.758
PU_3	Perceived usefulness	0.167	0.017	0.612

Extraction method: maximum likelihood. Rotation method: varimax with Kaiser normalization.

As shown in Table 2, the analysis results were perfectly consistent with the theoretical constructs: all items showed high loadings (>0.6) on their expected factors and extremely low cross-loadings on other factors. This indicates good discriminant validity among the three constructs. In summary, the EFA results provide strong evidence for the construct validity of the measurement tools used in this study.

#### 3.3.4 Control variables

Considering that gender and major background might influence attitudes toward new technologies, this study included them as control variables. Additionally, according to TAM theory, Perceived Usefulness is the strongest predictor of intention to use (Davis, 1989), so this variable was also measured and controlled.

### 3.4 Data analysis strategy

Data analysis was performed using the Python 3.11 programming environment, primarily utilizing the pandas (for data manipulation), statsmodels (for statistical modeling), and matplotlib/seaborn (for visualization) libraries. The specific analysis steps were as follows:

Common Method Bias Test: Harman’s single-factor test was used to assess the presence of serious common method bias.

Descriptive Statistics and Reliability Analysis: Means, standard deviations, and Cronbach’s *α* coefficients were calculated for each variable.

Correlation Analysis: Pearson correlation coefficients were used to examine the bivariate relationships among the main variables.

Hypothesis Testing:

H1 Test: Hierarchical linear regression was employed. Control variables were entered in the first step, followed by the independent variable (depression level) in the second step, observing the change in R^2^ and the significance of the regression coefficient.

H2 Test: A moderation effect model was used. A regression equation including the interaction term (Depression × Privacy Concern) was constructed. To avoid multicollinearity, the independent and moderator variables were mean-centered before creating the interaction term.

Simple Slope Analysis: A moderation effect plot was created to test the effect of depression on intention to use at low (−1 SD) and high (+1 SD) levels of privacy concern.

## 4 Results

### 4.1 Common method bias and normality tests

Since all variables in this study were collected from the same subjects at a single point in time through self-report measures, common method bias (CMB) could be a potential issue. The results of Harman’s single-factor test showed that there were 4 factors with eigenvalues greater than 1, and the first common factor explained a variance far below the critical threshold of 50%. This indicates that there are no serious common method bias issues in this study’s data.

### 4.2 Descriptive statistics and correlations

Before hypothesis testing, this study first conducted reliability analysis, descriptive statistics, and correlation analysis on the core variables to ensure data reliability and the validity of subsequent analyses.

#### 4.2.1 Reliability analysis

As shown in Table 3, the Cronbach’s *α* coefficients for all scales exceeded the acceptable standard of 0.7. The internal consistency of the Depression scale (*α* = 0.888) and the Intention to Use scale (*α* = 0.875) reached excellent levels, indicating that the measurement tools used in this study have good reliability.

**Table 3:** Results of reliability analysis.

Variable	Cronbach’s alpha (*α*)
Depression (PHQ-8)	0.888
Privacy concern	0.792
Perceived usefulness	0.728
Intention to use	0.875

#### 4.2.2 Descriptive statistics and normality test

This study conducted descriptive statistical analysis on the core variables, with results shown in Table 4. It is noteworthy that the average total score for the sample on the PHQ-8 depression scale (0–24 point scale) was 6.07 (SD = 4.60). According to the standard clinical cutoffs for the PHQ-8, the average level of this university student sample falls within the “mild depression” range.

**Table 4:** Descriptive statistics for key variables (*N* = 612).

Variable	Mean	SD	Min	Max	Skewness	Kurtosis
Depression (PHQ-8 score, 0–24)	6.07	4.60	0.00	21.00	0.62	−0.32
Privacy concern (1–5)	3.41	0.94	1.00	5.00	−0.24	−0.56
Perceived usefulness (1–5)	3.57	0.77	1.00	5.00	−0.29	−0.36
Intention to use (1–5)	3.36	0.91	1.00	5.00	−0.13	−0.57

**Figure 2:**
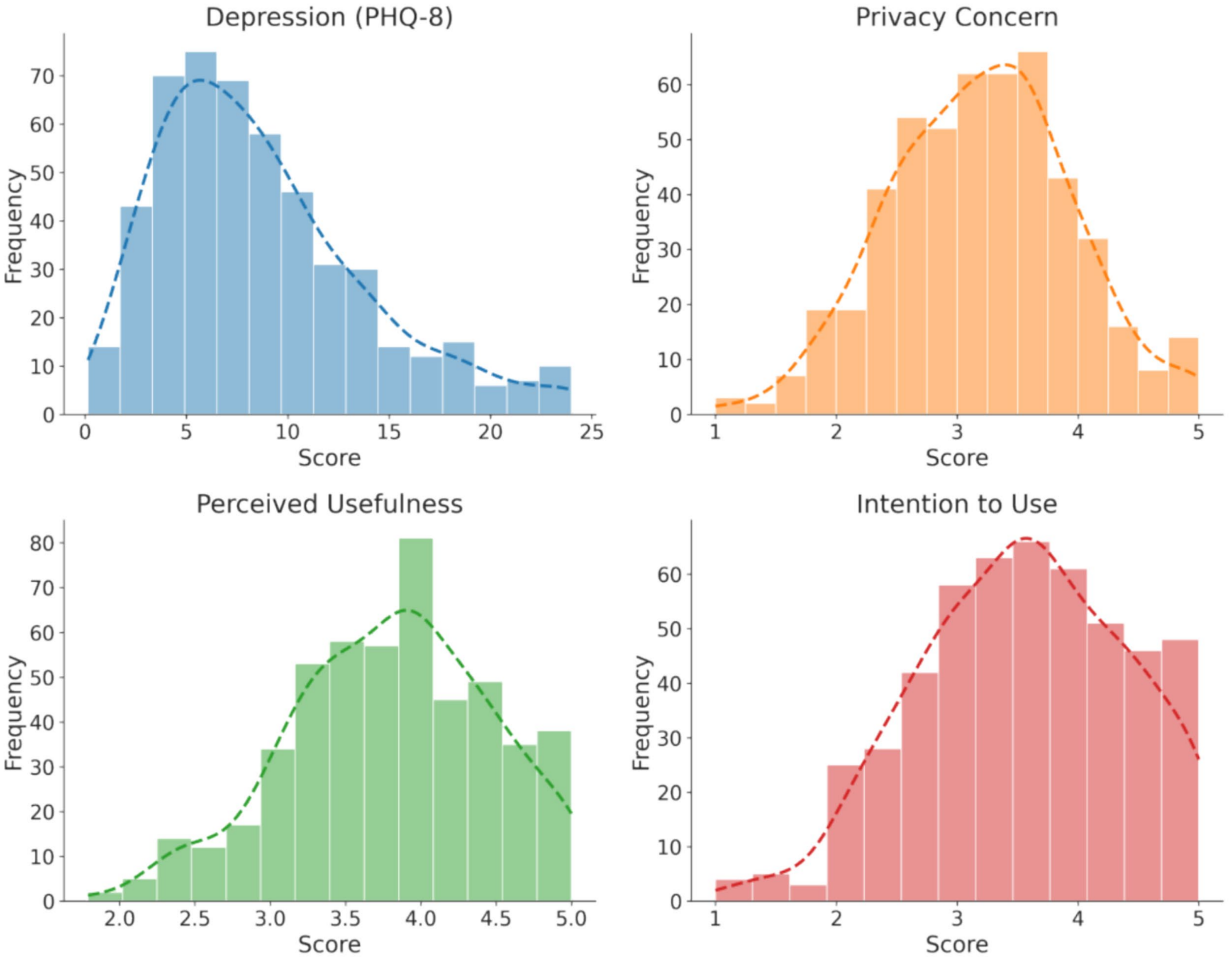
Distribution histograms and kernel density curves of core variables.

To test for data normality, this study combined numerical and graphical tests. Numerically, Table 4 shows that the absolute values of skewness and kurtosis for all variables are less than 1, which is well below the recommended threshold of 2. Graphically, the histograms in Figure 2 provide further visual evidence, showing that the distributions of all variables are well-shaped and approximate a normal distribution. In summary, the data distribution meets the prerequisite conditions for subsequent Pearson correlation analysis and multiple regression analysis.

#### 4.2.3 Correlation analysis

**Figure 3:**
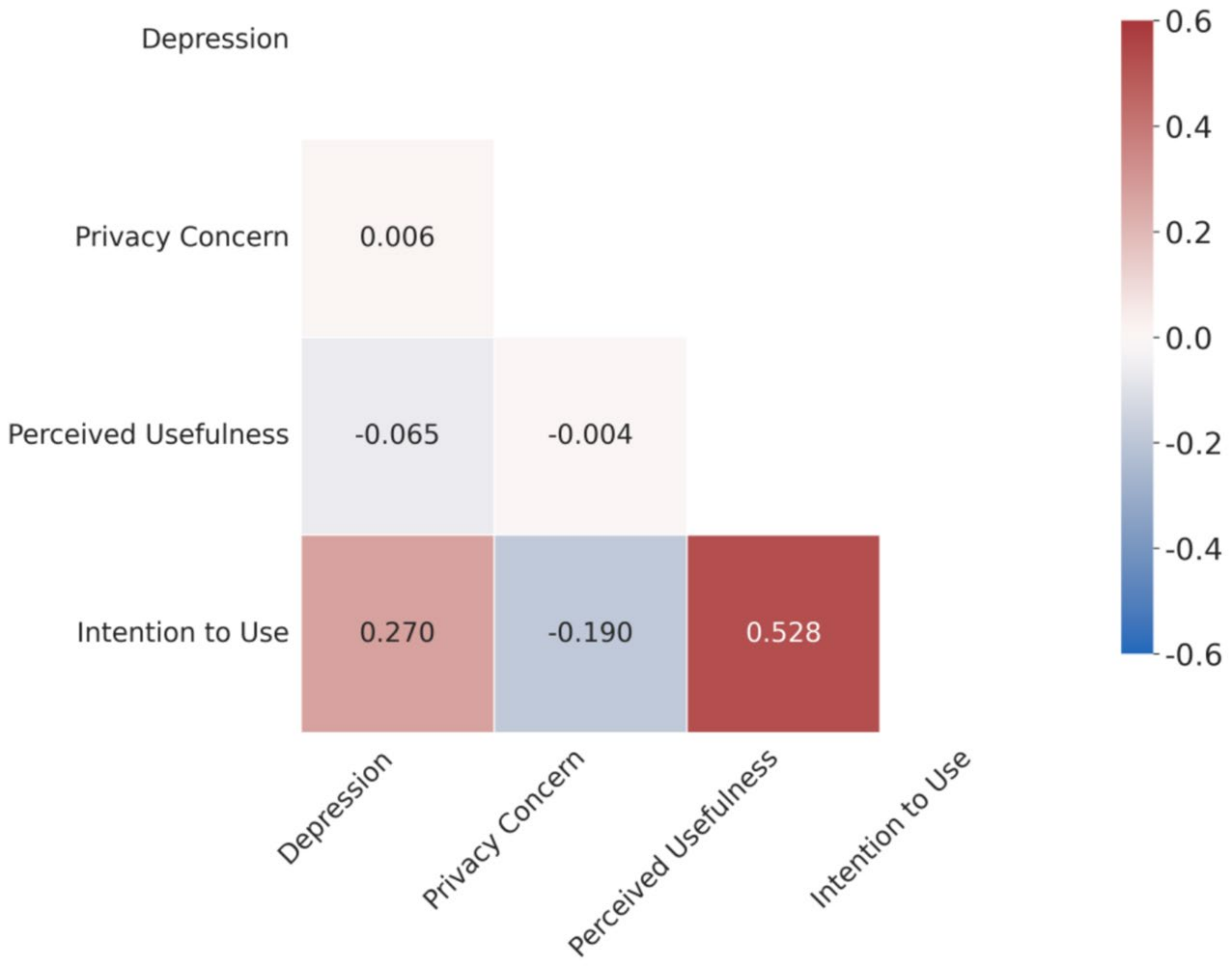
Heatmap of the Pearson correlation matrix.

The Pearson correlation coefficient matrix among the variables is shown in Table 5. To more intuitively display the relationships between variables, we further plotted a correlation heatmap (Figure 3).

**Table 5:** Pearson correlation matrix for key variables.

Variable	1. Depression	2. Privacy concern	3. Perceived usefulness	4. Intention to use
1. Depression	1			
2. Privacy concern	0.006	1		
3. Perceived usefulness	−0.065	−0.004	1	
4. Intention to use	0.270**	−0.190*	0.528*	1

*N* = 612. **p < 0.01.

The results clearly show that depression level is significantly positively correlated with intention to use (*r* = 0.270, *p* < 0.01), meaning that students with higher levels of depression have a stronger intention to use AI music therapy. Privacy concern is significantly negatively correlated with intention to use (*r = −0.190, p < 0.01*), indicating that students more concerned about privacy have a lower intention to use. Perceived usefulness has the strongest positive correlation with intention to use (*r* = 0.528, *p* < 0.01), which is highly consistent with the classic theory of the Technology Acceptance Model (TAM) (Davis, 1989) and also validates the effectiveness of the measurement tools in this study. These results provide strong preliminary evidence for the subsequent hypothesis testing.

### 4.3 Hypothesis testing

To test the hypotheses proposed in this study, we employed hierarchical multiple regression analysis. The detailed results are presented in Table 6. This table clearly displays the analysis data for three models used to validate our research hypotheses.

**Table 6:** Results of hierarchical multiple regression analysis predicting intention to use.

Variable	Model 1		Model 2		Model 3	
	*B* (SE)	*β*	*B* (SE)	*β*	*B* (SE)	*β*
Step 1: control variables						
(Constant)	0.980 (0.165)		0.978 (0.162)		0.999 (0.162)	
Gender (female = 1)	0.016 (0.071)	0.009	0.010 (0.070)	0.006	0.016 (0.070)	0.009
Major (vs. STEM)						
Humanities and Social Sci.	0.034 (0.078)	0.017	0.035 (0.077)	0.017	0.034 (0.077)	0.017
Medicine	0.041 (0.079)	0.020	0.043 (0.078)	0.021	0.045 (0.078)	0.022
Arts	0.002 (0.080)	0.001	0.003 (0.079)	0.002	0.002 (0.079)	0.001
Perceived usefulness	0.623 (0.040)	0.528***	0.605 (0.040)	0.513***	0.605 (0.040)	0.513***
Step 2: independent variable						
Depression (centered)			0.025 (0.007)	0.128***	0.026 (0.007)	0.133***
Step 3: moderator and interaction						
Privacy concern (centered)					−0.030 (0.037)	−0.031
Depression × privacy concern					−0.017 (0.007)	−0.086*
Model summary						
*R* ^2^	0.305		0.320		0.327	
Adjusted *R*^2^	0.300		0.313		0.318	
*ΔR^2^*			0.015		0.007	
*F*-test	52.87***		47.38***		41.52***	
*ΔF*			11.21**		5.92*	

*N* = 612. For brevity, only standardized coefficients (*β*) are shown. The independent variable (depression) and moderator (privacy concern) were mean-centered.

*p < 0.05, **p < 0.01, ***p < 0.001.

#### 4.3.1 Testing hypothesis H1: the main effect of depression on intention to use

Hypothesis H1 predicted that university students’ level of depression would positively influence their intention to use AI music therapy.

In Model 1 of Table 6, we first introduced the control variables (gender, major, perceived usefulness). The results showed that this model could explain 30.5% of the total variance in intention to use ( R2 = 0.305).

Subsequently, in Model 2, we added the independent variable “Depression Level” to Model 1. The results showed that depression level had a significant positive predictive effect on intention to use (*β = 0.128, p < 0.001*). The introduction of this variable increased the model’s overall explanatory power from 30.5 to 32.0%, and the change in *R*^2^ ( Δ *R*^2^ = 0.015) was highly statistically significant (F-change = 11.21, *p < 0.01*). Furthermore, an examination of the model’s residuals indicated that they were evenly distributed around 0 with no obvious heteroscedasticity (see Supplementary material B), satisfying the basic assumptions of a linear regression model.

**Figure 4:**
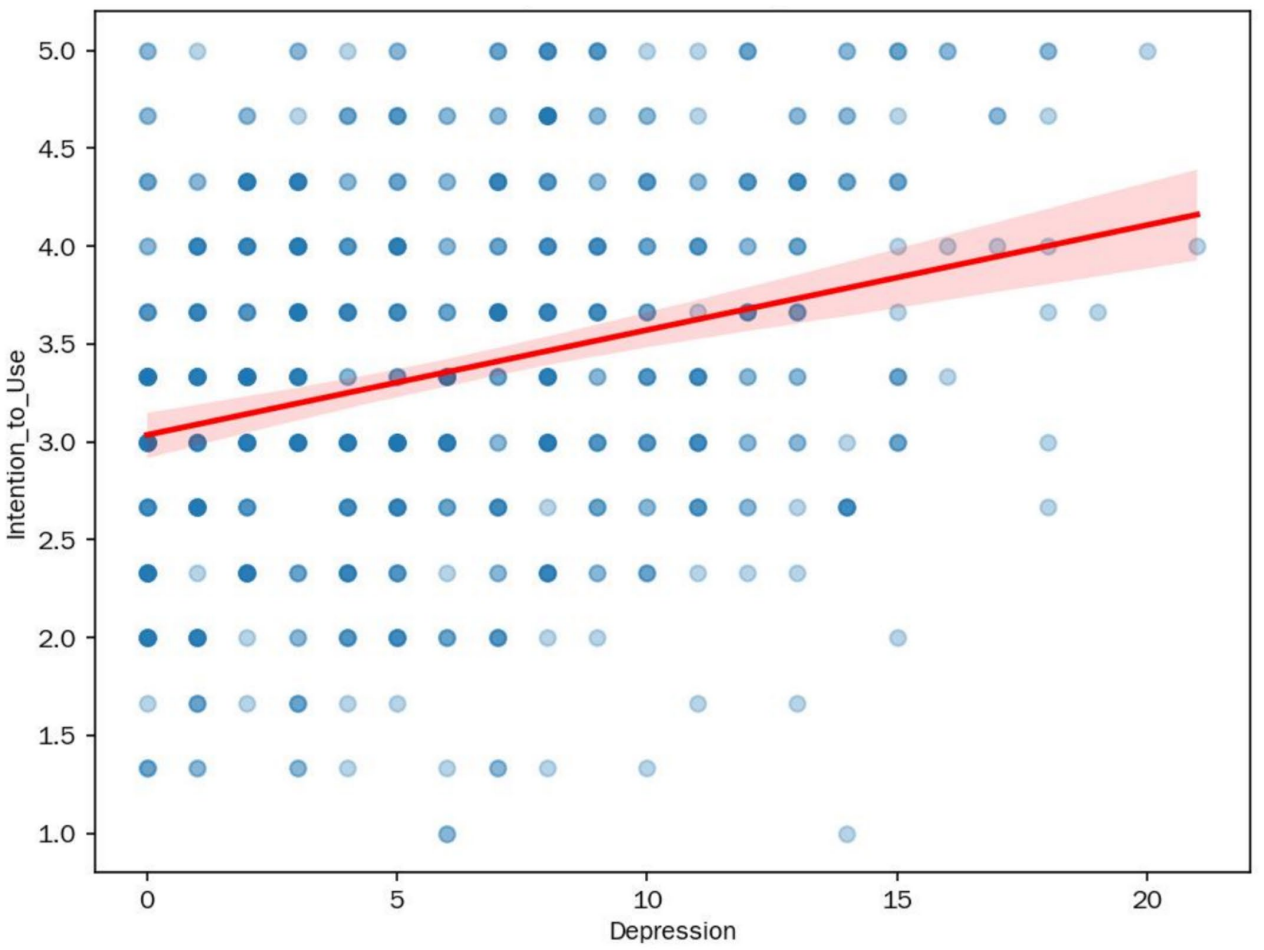
Scatter plot of depression and intention to use.

This positive relationship is intuitively demonstrated in the scatter plot in Figure 4: the overall trend of the data points indicates that as an individual’s depression score increases, their intention to use the AI music therapy tool also strengthens.

In summary, the regression analysis results provide strong support for Hypothesis H1.

#### 4.3.2 Testing hypothesis H2: the moderating role of privacy concerns

Hypothesis H2 predicted that privacy concerns would play a negative moderating role in the relationship between depression and intention to use.

To test this hypothesis, we constructed Model 3, which included the interaction term. As shown in Table 6, after introducing the interaction term (Depression × Privacy Concern), its regression coefficient was significantly negative (*β = −0.086, p < 0.05*). This indicates that the level of privacy concern indeed significantly alters the strength of the effect of depression on intention to use.

**Figure 5:**
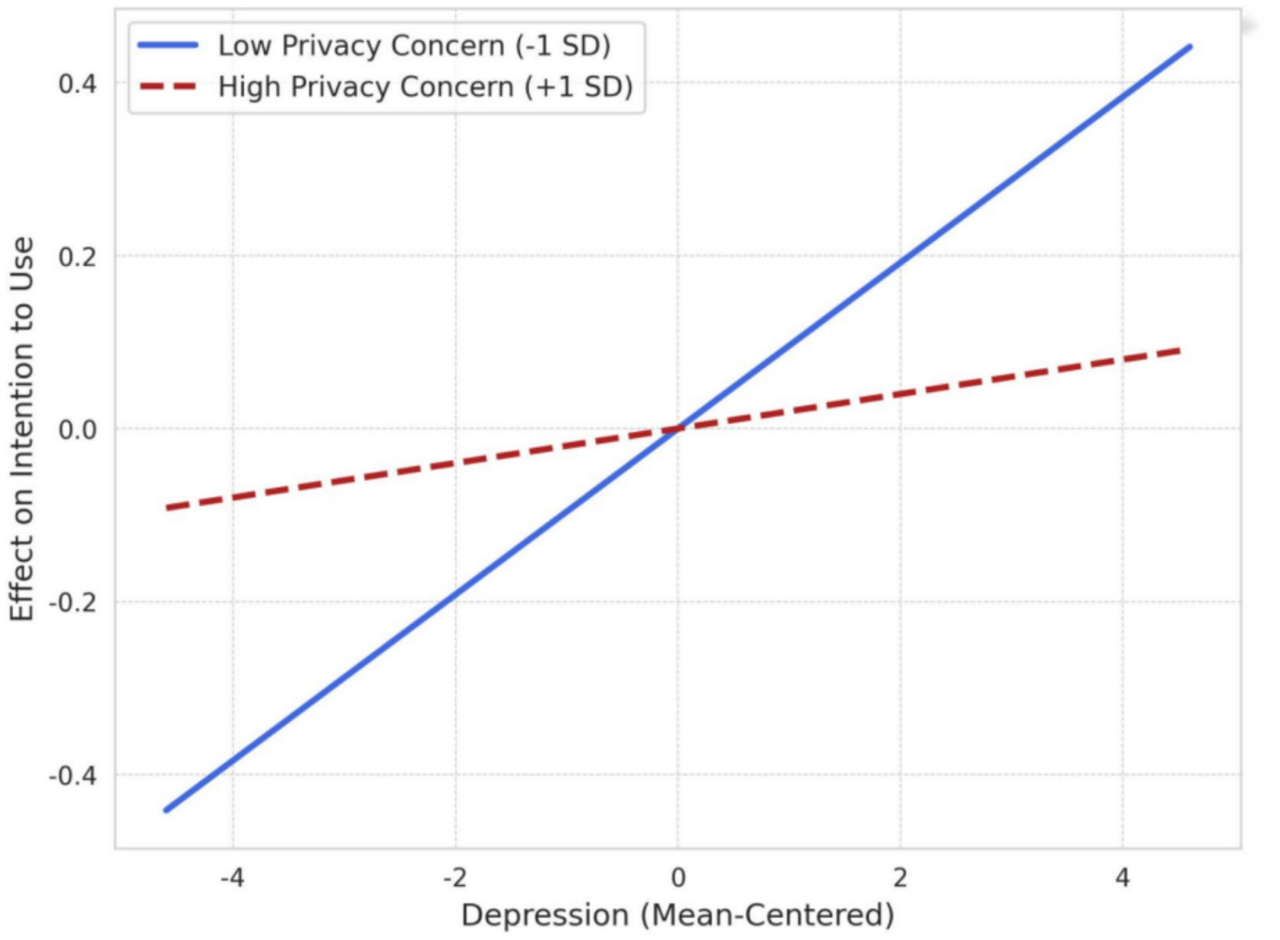
Moderation effect of privacy concern on the relationship between depression and intention to use.

To more clearly explain this interaction effect, we conducted a simple slope analysis and plotted the moderation effect (Figure 5).

Low Privacy Concern Group (−1 SD): As shown by the blue line, for students who were less concerned about privacy breaches, the positive predictive effect of depression level on intention to use was strong and significant (simple slope *B* = 0.041, *p < 0.001*). In this case, their emotional distress effectively translated into a motivation to seek AI-powered help.

High Privacy Concern Group (+1 SD): As shown by the red line, for students highly concerned about their privacy, the positive predictive effect of depression level on intention to use became flat and was no longer significant (simple slope *B* = 0.010, *p = 0.312*). This suggests that strong privacy concerns “inhibited” the help-seeking motivation; even if they had high levels of depressive mood, this mood did not effectively translate into an intention to use.

In summary, the results of the simple slope analysis clearly demonstrate the negative moderating effect of privacy concerns. Hypothesis H2 is fully supported.

## 5 Discussion and conclusion

### 5.1 Discussion of findings

Based on real-world data (*N* = 612), this study delved into the complex relationship between depressive mood and the acceptance of AI music therapy. The results not only support the basic logic of the association between distress and help-seeking but also highlight the crucial moderating role of ‘privacy concerns’ as a key braking mechanism.

#### 5.1.1 The “digital help-seeking” tendency of depressed groups

The study found that after controlling for variables such as perceived usefulness, depression level still significantly and positively predicted university students’ intention to use AI music therapy (*β = 0.128*). This finding is consistent with the theoretical expectations of the ‘Deficiency Compensation Hypothesis,’ which posits that when individuals’ needs for emotional support are not met in reality, they may turn to low-social-pressure online environments for alternative satisfaction. Unlike previous research suggesting that depression leads to behavioral withdrawal, this study shows that when faced with non-interpersonal digital tools, depressed individuals are associated with a stronger approach motivation. This aligns with findings from some studies that students with mental health needs show a higher intention to use digital mental health solutions like apps and chatbots (Gbollie et al., 2023; Sun et al., 2025). The psychological mechanism behind this phenomenon may stem from the intrinsic nature of AI music therapy as a non-interpersonal interactive tool. Specifically, the ‘non-judgmental space’ it provides can precisely bypass the social evaluation and stigma concerns that may exist in traditional psychological help-seeking (Li et al., 2024). This is perhaps the key to its appeal to depressed individuals, and it also enables it to be an effective supplement to mental health services (Harith et al., 2022).

#### 5.1.2 The “chilling effect” of privacy concerns

The most central finding of this study is the revelation of the significant moderating role of privacy concerns on the relationship between distress and help-seeking intention (H2). The interaction plot (Figure 5) clearly illustrates the “help-seeking vs. privacy” trade-off: in the low privacy concern group (blue line), the positive effect of depression on intention to use is very strong. However, in the high privacy concern group (red line), this positive pathway, while still present, is significantly weakened.

This statistical “chilling effect” provides a possible explanatory framework for understanding the real-world dilemma of why many depressed students, despite knowing they need help, hesitate to use mental health apps. For those depressed individuals with high privacy sensitivity, the fear of data leakage may override the need to seek comfort, thereby potentially being associated with a diminished intention to seek help. For depressed individuals, their psychological defenses are already fragile; they not only fear being spied on but also dread the social consequences of data leakage (such as being discriminated against). When “privacy anxiety” surpasses the “need for therapy,” they fall into a predicament of “the more pain, the more silence.” This is a specific manifestation of the “privacy paradox” in the mental health field (Barth and de Jong, 2017). It is also important to note that while the moderation effect is statistically significant and theoretically meaningful, its effect size is modest (*β* = −0.086). This is common in behavioral research where multiple factors influence intentions, but it underscores that privacy concern is one of several important factors in this complex decision-making process.

### 5.2 Theoretical implications

First, this study expands the boundaries of the Technology Acceptance Model (TAM) in a psychopathological context. By introducing the variable “depression,” it demonstrates that in the health domain, users’ decision-making logic is not only “rationality-driven” but also “emotion-driven.” Users in a depressive state are not merely calculating “efficiency” but are weighing “a sense of security.” This finding offers a new perspective on the application of the TAM framework to specific populations and in the context of AI, echoing the research trend of integrating user traits, ethics, and trust into TAM (Mustofa et al., 2025; Zhang et al., 2025).

Second, it provides new empirical evidence for Privacy Calculus Theory. This study confirms that the privacy sensitivity of depressed groups is extremely high, with their perceived risk weight being far greater than that of ordinary users. This indicates that in privacy research on special populations, the psychological traits of individuals must be considered, which is in line with research validating privacy calculus in other sensitive contexts (such as location-based services) (Xu et al., 2009).

### 5.3 Practical implications

For University Counseling Centers:

Build Trust Mechanisms: When promoting AI tools, it is essential to explicitly promise that “data will not be entered into official records” and “data will be stored locally only.” Only by alleviating these concerns (reducing privacy fears) can the benefits of technology reach those depressed students with high privacy sensitivity.

Tiered Intervention: For students at high risk of severe depression, simply recommending technological tools may be ineffective. It needs to be coupled with privacy security education or providing completely offline usage options. The views and recommendations of mental health professionals are also crucial (Sharif et al., 2025).

For AI Product Developers:

Privacy by Design: Adopt technologies like federated learning or on-device computing to ensure data does not leave the local device from the foundational technical level.

Empathetic Privacy Interaction: Use warm, straightforward language instead of cold legal clauses to inform users that their data is safe, thereby lowering their psychological defenses.

Ethical Safeguards & Escalation Paths: Developing AI tools should not solely pursue algorithmic precision but must also anticipate the possibility of “making mistakes.” An ethical safety net should be established. For example:

Crisis Identification and Intervention: When the algorithm identifies serious psychological crisis signals (such as suicidal ideation) from user input (e.g., text, voice), the system must not just provide a piece of music. It must immediately suspend routine interaction and provide a clear, prominent emergency intervention portal, such as a one-click call to a mental health support hotline or an emergency contact.

“Human-in-the-Loop” Design: The system should offer a seamless option to transfer to a human counselor, ensuring that users can get timely support from a real person when they feel the AI cannot meet their needs or causes discomfort. This reflects the fundamental principle of “tech for good,” where AI should serve as an aid to human experts, not a complete replacement.

For School Health Policy Makers: University administrators play a pivotal role in the adoption of ecosystem-level digital tools. It is recommended that universities establish a digital ethics review committee to vet third-party mental health apps before officially recommending them to students. Furthermore, schools should promote “Digital Health Literacy” education, helping students understand how to identify secure apps that genuinely protect their mental health data, thereby reducing irrational privacy fears and fostering a safer campus digital environment.

### 5.4 Limitations and future directions

Although this study has yielded valuable conclusions, it still has some limitations, which also point to directions for future research:

Causal Inference Limitations of Cross-Sectional Design: This study used a cross-sectional design, which can only reveal correlations between variables, not establish strict causal chains. Although our model posits that depression predicts the intention to use, the possibility of reverse causality cannot be ruled out—that is, individuals who are already more open to digital therapies and have a higher intention to seek help may also be more willing to report their depressive symptoms in a questionnaire. Future research should adopt longitudinal designs or experimental methods (such as randomized controlled trials) to more clearly reveal the causal relationships through tracking over multiple time points or experimental interventions (Fitzpatrick et al., 2017; Liu et al., 2025).

Selection Bias from Sampling Method: This study used a combination of convenience and snowball sampling, which inevitably introduces the risk of self-selection bias. Recruiting through online questionnaire links likely attracted specific types of students: first, students more interested in and proficient with emerging technologies like AI may have been more willing to participate (Abbas et al., 2024); second, students with a more open attitude toward mental health topics and less stigma may also have been more inclined to complete the questionnaire. These two biases may have jointly led to an overestimation of the acceptance (intention to use) of AI music therapy in the sample. This could also make the association between depression and help-seeking intention appear stronger in our sample than in the general population. Therefore, caution is needed when generalizing the findings of this study to the entire university student population. Future research should strive to use probability sampling methods, such as stratified random sampling, to obtain more representative samples and enhance the external validity of the conclusions.

Self-Report Data Bias: All data in this study were collected through participant self-reports and may be subject to social desirability effects. Although we provided anonymity guarantees, participants might still unconsciously hide their true level of privacy concern or exaggerate their help-seeking intentions. To obtain more objective insights, future research could combine backend behavioral data (such as actual app download rates and usage duration) or physiological measures (such as galvanic skin response to assess emotional arousal) for multi-source data cross-validation (Kelly et al., 2022).

Abstract nature of the construct measurement: this study defined “AI music therapy” through a textual description, measuring participants’ intention to accept a “conceptual” tool. However, different participants’ imaginations of this concept could vary greatly (from an “intelligent playlist” to “brain-computer interface music generation”), and this heterogeneity in imagination might introduce noise into the measurement. Future research could enhance ecological validity by providing more concrete stimuli, for example, by having all subjects watch a standardized operational video of a real AI music therapy app, or even allowing them to briefly try it out before completing the questionnaire.

Single Cultural Context: The sample for this study was primarily from universities in mainland China. Therefore, the conclusions may be influenced by specific cultural contexts, such as stigma in a collectivist culture, and trust in authority and official platforms (He et al., 2024; Li et al., 2025; Qi et al., 2025). In different cultural backgrounds, the “help-seeking vs. privacy” trade-off and its manifestations may differ. Thus, it is necessary to conduct cross-cultural comparative studies in the future to test the generalizability of this study’s model and explore the role of cultural factors.

## 6 Conclusion

In the digital wave, AI brings infinite possibilities to mental health services (Ni and Jia, 2025), but it also casts a shadow of privacy. This study indicates that depression acts as a significant positive predictor for university students’ intention to embrace AI music therapy, while simultaneously being associated with heightened privacy sensitivity. Therefore, the findings suggest that the key to resolving this core contradiction may lie not solely in developing smarter algorithms (Oleribe et al., 2025), but in building a more trustworthy wall of privacy protection. Only when technology genuinely gives users a sense of security can the digital support it provides effectively reach and help those individuals in psychological distress.

## Data Availability

The original contributions presented in the study are included in the article/Supplementary material, further inquiries can be directed to the corresponding author.
