# Supplementary material for: The conflict between need and fear: how privacy concerns moderate the influence of depression on university students’ acceptance of AI music therapy
Source: Front Psychol. 2026 Feb 24;17:1768759. doi: 10.3389/fpsyg.2026.1768759 (PMC12971948; doi:10.3389/fpsyg.2026.1768759)
Supplement: Supplementary file 1 [file Data_Sheet_1.zip › Appendix A .docx]

### **Appendix A: Research Questionnaire**

**Part I: Informed Consent and Basic Information**

Welcome to this survey! This research aims to understand the views and usage intentions of university students regarding “AI Music Emotion Regulation” technology. All data will be used for academic statistical analysis only.

This survey is completely anonymous. We will not collect any personally identifiable information (such as name, student ID, IP address, etc.). The content of this questionnaire does not involve sensitive privacy, and the risk to you is extremely low. Participation is entirely voluntary, and you may stop the survey at any time. This study is only for university students who are 18 years of age or older.

If you have read and understood the above information and agree to participate in this study, please proceed.

**1.Your gender:**

□ Male □ Female

**2.Your year of birth:** __________

**3.Your current academic level:**

□ Undergraduate □ Master’s student □ PhD student

**4.Your field of study:**

□ STEM □ Humanities & Social Sciences □ Medicine/Psychology □ Arts/Sports □ Other

**5.How would you describe your family’s financial situation?**

□ Difficult □ Lower-middle □ Middle □ Upper-middle □ Affluent

**6.How often do you listen to music on your phone?**

□ Almost never □ Occasionally (1-2 times a week) □ Frequently (3-5 times a week) □ Every day

**7.Have you ever used any “mental health/sleep aid/meditation” apps?**

□ Never heard of them □ Heard of them but never used □ Used them but stopped □ Currently using

**Part II: Recent State Assessment (PHQ-8)**

Instructions: Over the last 2 weeks, how often have you been bothered by any of the following problems?

| Item | Not at all (0) | Several days (1) | More than half the days (2) | Nearly every day (3) |
| --- | --- | --- | --- | --- |
| 8. Little interest or pleasure in doing things. | □ | □ | □ | □ |
| 9. Feeling down, depressed, or hopeless. | □ | □ | □ | □ |
| 10. Trouble falling or staying asleep, or sleeping too much. | □ | □ | □ | □ |
| 11. Feeling tired or having little energy. | □ | □ | □ | □ |
| 12. Poor appetite or overeating. | □ | □ | □ | □ |
| 13. Feeling bad about yourself—or that you are a failure or have let yourself or your family down. | □ | □ | □ | □ |
| 14. Trouble concentrating on things, such as reading the newspaper or watching television. | □ | □ | □ | □ |
| 15. Moving or speaking so slowly that other people could have noticed? Or the opposite—being so fidgety or restless that you have been moving around a lot more than usual. | □ | □ | □ | □ |

**Part III: Views on AI Music Therapy Tools**

**[Scenario Description]** Please imagine a mobile app: it’s an “AI Emotion Regulation Assistant” that requires no human counselors. When you feel stressed or emotionally unwell, it can analyze your state (e.g., voice, heart rate, or self-report) through an AI algorithm and automatically generate a personalized piece of soothing music for you, aiming to help you relax.

**Instructions:** Based on the description above, please rate your level of agreement with the following statements.

| Dimension | Item | Strongly Disagree (1) | Disagree (2) | Neutral (3) | Agree (4) | Strongly Agree (5) |
| --- | --- | --- | --- | --- | --- | --- |
| Perceived Usefulness | 16. I think this AI tool could help me relieve psychological stress. | □ | □ | □ | □ | □ |
|  | 17. Compared to talking to someone, using music to regulate my emotions is more efficient for me. | □ | □ | □ | □ | □ |
|  | 18. Overall, I think this technology would be useful for my mental health. | □ | □ | □ | □ | □ |
| Perceived Ease of Use | 19. I think it would be easy for me to learn how to use this AI tool. | □ | □ | □ | □ | □ |
|  | 20. I think using it on my phone anytime, anywhere would be convenient. | □ | □ | □ | □ | □ |
| Privacy Concern | 21. I am concerned that this AI tool would excessively collect my emotional data. | □ | □ | □ | □ | □ |
|  | 22. I am concerned that my mental health data could be disclosed to the university or third parties. | □ | □ | □ | □ | □ |
|  | 23. I would hesitate to use this tool because of privacy and security concerns. | □ | □ | □ | □ | □ |
| Technology Trust | 24. I believe the AI algorithm could accurately judge my current emotional state. | □ | □ | □ | □ | □ |
|  | 25. I believe the music generated by the AI is safe and has no side effects for me. | □ | □ | □ | □ | □ |
| Intention to Use | 26. If I had the opportunity, I would be willing to try this AI music tool. | □ | □ | □ | □ | □ |
|  | 27. When I feel down, I would consider using this tool. | □ | □ | □ | □ | □ |
|  | 28. I would recommend this type of tool to classmates or friends in need. | □ | □ | □ | □ | □ |

This is the end of the questionnaire. Thank you very much for your valuable time and contribution!

*This questionnaire is for statistical research purposes only. Occasional emotional fluctuations are normal, but if you find yourself struggling with your emotions for a prolonged period, please remember you are not alone, and seeking professional support is an effective way to solve problems. National Psychological Assistance Hotline: 400-161-9995.*
